# Interactions within the microbiome alter microbial interactions with host chemical defences and affect disease in a marine holobiont

**DOI:** 10.1038/s41598-018-37062-z

**Published:** 2019-02-04

**Authors:** Sharon R. Longford, Alexandra H. Campbell, Shaun Nielsen, Rebecca J. Case, Staffan Kjelleberg, Peter D. Steinberg

**Affiliations:** 1 Centre for Marine Bio-Innovation, and School of Biological, Earth and Environmental Sciences, University of New South Wales, Sydney, NSW 2052 Australia; 2 Singapore Centre for Environmental Life Sciences Engineering, 60 Nanyang Drive, SBS-01N-27, Singapore, 637551 Singapore; 3 Sydney Institute of Marine Science, Chowder Bay Road, Mosman, NSW 2088 Australia; 4 GeneCology Research Centre, University of the Sunshine Coast, Sippy Downs, QLD 4556 Australia; 5 Department of Biological Sciences, Biological Sciences Centre, University of Alberta, Edmonton, Alberta T6G 2E9 Canada; 6 School of Biological Sciences, Nanyang Technological University, 60 Nanyang Drive, Singapore, 637551 Singapore

## Abstract

Our understanding of diseases has been transformed by the realisation that people are holobionts, comprised of a host and its associated microbiome(s). Disease can also have devastating effects on populations of marine organisms, including dominant habitat formers such as seaweed holobionts. However, we know very little about how interactions between microorganisms within microbiomes - of humans or marine organisms – affect host health and there is no underpinning theoretical framework for exploring this. We applied ecological models of succession to bacterial communities to understand how interactions within a seaweed microbiome affect the host. We observed succession of surface microbiomes on the red seaweed *Delisea pulchra in situ*, following a disturbance, with communities ‘recovering’ to resemble undisturbed states after only 12 days. Further, if this recovery was perturbed, a bleaching disease previously described for this seaweed developed. Early successional strains of bacteria protected the host from colonisation by a pathogenic, later successional strain. Host chemical defences also prevented disease, such that within-microbiome interactions were most important when the host’s chemical defences were inhibited. This is the first experimental evidence that interactions within microbiomes have important implications for host health and disease in a dominant marine habitat-forming organism.

## Introduction

Disease is emerging as a fundamentally important factor affecting the ecology and evolution of higher organisms by exerting profound effects on host performance, fitness and/or survival^1–3^. Disease frequency and severity are predicted to increase as environments change, due both to impacts on host reliance^4^ and on pathogen abundance, behavior or virulence^5–7^. However, our understanding of microbial disease is undergoing a paradigm shift *via* the holobiont approach and both the classical concept of disease in which a single pathogen infects a single host^8^ and the traditional methodology for confirming the presence of a disease via the application of Koch’s postulates^9^, are now being challenged. The holobiont approach involves assessing the health of a host organism in the context of its associated microbiome(s)^10,11^ and studies supporting the interdependence of macro- and microbiota within holobionts have recently emerged for diverse organisms including humans^11^ and demonstrate a clear association between shifts in the composition of microbiomes and disease in the host^12–15^.

These observations imply that interactions within microbiomes are important for their hosts. However, we are only just beginning to explore interactions between microorganisms within microbiomes and we still understand very little about the mechanisms by which such interactions may lead to disease in the host (but see^16–18^). One means to help understand the role of microbial interactions on hosts is to draw upon existing eukaryotic ecological theory, and its long history of theoretical frameworks for understanding interactions within and among communities. Microbiomes are communities embedded in an ecosystem (the host and the broader environment), so the application of theoretical community ecology to microbial communities can be a useful way to guide examinations into microbiomes^16–18^ and how their interactions could influence host health. Furthermore, microbial communities are arguably ideal systems for testing ecological theory, because of their relatively short generation times (both in an absolute sense and relative to their eukaryotic hosts), which allow equilibrium states to be achieved over short periods of time (as little as 20 min^19^); and their suitability for well-replicated, community-scale manipulations without concern for large-scale impacts on sensitive, natural environments.

Here we used succession theory to investigate interactions within the microbiome of a seaweed – the dominant habitat-forming organisms on temperate reefs. Ecological succession is the process of post-disturbance community change and a fundamental concept within the field of community ecology. Since its formalization more than a century ago^20^, ecological succession has been the focus of much theoretical and experimental work e.g.^21–24^ and can be defined as a directional, continuous and non-seasonal pattern of population colonization and extinction in a defined spatial area^25^. Various conceptual approaches to succession in ecological systems have been adopted, but one useful approach is that of Connell & Slatyer^21^, who proposed three alternative models of succession – facilitation, tolerance and inhibition, which are differentiated based on whether initial or ‘early successional species’ (ESS) have positive (facilitation), neutral (tolerance) or negative (inhibition) impacts on later successional species (LSS).

Recently, some studies have investigated how microbial communities change over time, however these have mostly been limited to human^26^, agricultural^27^ or controlled laboratory^28^ settings with very few examples of experimental manipulations of microbiomes under natural conditions (but see^17,18^). Furthermore, when the importance of priority effects on bacterial colonization^17^ and diversity on dispersal^18^ have been experimentally assessed, investigations have not been extended towards understanding how micro-micro interactions within microbiomes affect host health, survival or performance. Here, we apply succession theory to understand how microbiomes associated with naturally occurring macroalgae change following an experimental disturbance *in situ* and whether interactions between microorganisms within microbiomes can affect disease incidence or severity in the host.

*Delisea pulchra* is a chemically defended red macroalga that produces halogenated furanones^29,30^, secondary metabolites that interfere with cell-cell signaling systems in many bacteria^31,32^. Halogenated furanones also protect *D. pulchra* from natural enemies, including epibiota^33^, herbivores^34^ and some microbial pathogens^32^ and their production can be manipulated *in vitro* via the removal of certain compounds from growth media^35,36^. When water temperatures are elevated and tissue concentrations of halogenated furanones are relatively low, this species suffers from a bacterial bleaching disease, which can affect more than 50% of individuals in populations at peak times^35^ and has severe consequences for affected individuals, including dramatic reductions in fecundity and growth, and altered interactions with consumers^37^.

This background understanding of host-microbe interactions in this seaweed holobiont, makes it an ideal system for investigations of within-microbiome interactions and their impacts on the host. In addition to within-microbiome interactions, *D. pulchra*’s chemical defences are a potential mechanism by which this host may influence the composition of its microbiome and potentially also, interactions within it. We experimentally disturbed microbiomes associated with replicate *D. pulchra* thalli, then replaced them in their natural environment and followed their re-development over time to see whether they showed evidence of predictable succession. In a second group of experiments, we also manipulated succession by inoculating experimental thalli with combinations of early- and late-successional microorganisms and assessed impacts of these microbiome manipulations on the development of symptoms of bleaching disease.

## Results

### Responses of *D. pulchra*’s microbiome to experimental disturbance

**Figure 1:**
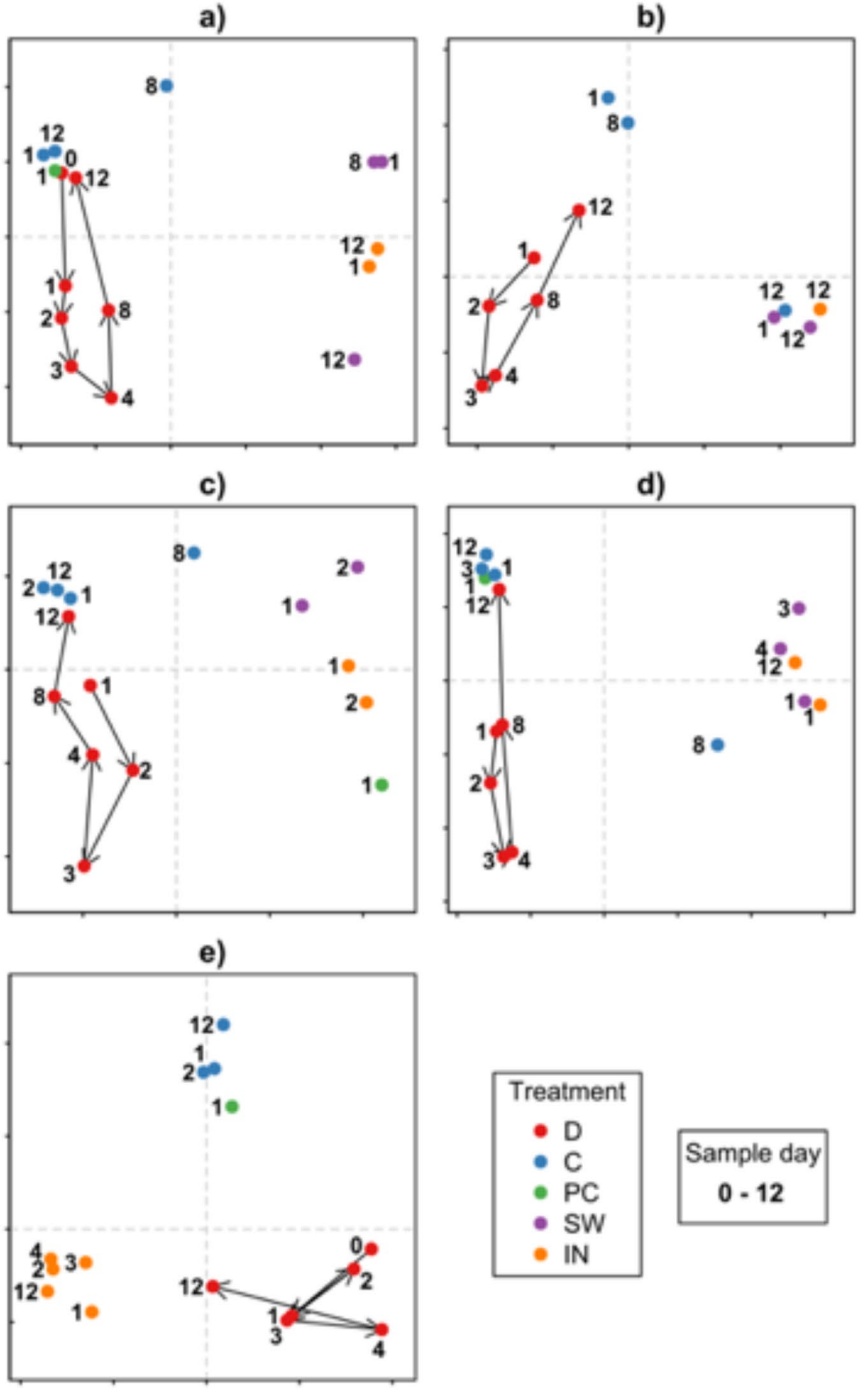
Principal component ordinations (PCOs) showing change in microbiomes associated with *D. pulchra* in experiments 1–5 (**a**–**e**) whose microbiomes were experimentally disturbed with antibiotic treatments (‘D’), procedural controlled (‘PC’) and unmanipulated (‘C’) controls, and microbiomes associated with the surrounding seawater (‘SW’) and those associated with inanimate surfaces (‘IN’, deployed at the same time as the experimental algae) over time (0–12 (**d**) as indicated by the number following the letter indicating treatment) during five independent ‘disturbance’ experiments. The hierarchical sampling scheme involved DGGE gel banding comparisons of replicates from each treatment (first level: n = 5–6; these yielded very low level between-replicate variability) and subsequent single-gel between-treatment comparison (second level: n = 1). Red arrows connect the sequential time points for the antibiotic treated *D. pulchra*. Variance explained (%) for each horizontal and vertical axis, respectively, are (**a**) 64, 19, (**b**) 54, 25, (**c**) 37,18 (**d**) 66, 33, (**e**) 37, 29.

Approximately 12 days after a significant experimental disturbance, *Delisea pulchra*’s microbiome reverted back to a composition similar to its initial community and those of ‘native’ (unmanipulated, *in situ*) microbiomes on co-occurring individuals. This pattern was observed repeatedly in four out of five independent experiments conducted over two years (Fig. 1). Data from experiment 1 were used to create a cyclic model of community shifts and microbial community changes in the disturbed *D. pulchra* treatments in subsequent experiments were tested against this model (three out of five times p ≤ 0.05; Fig. 1; Table 1). In experiment 3, a clear cyclic pattern was observed although it failed to match the cyclic model statistically due to different rates of succession (recovery of the microbiome was either faster or slower in this experiment than the others). Despite the poor fit in this instance, the cyclic shift is clearly evident in the nMDS plot from this experiment and overall, cyclic change in microbial communities was consistently observed among the disturbed *D. pulchra* treatments in all experiments.

**Figure 2:**
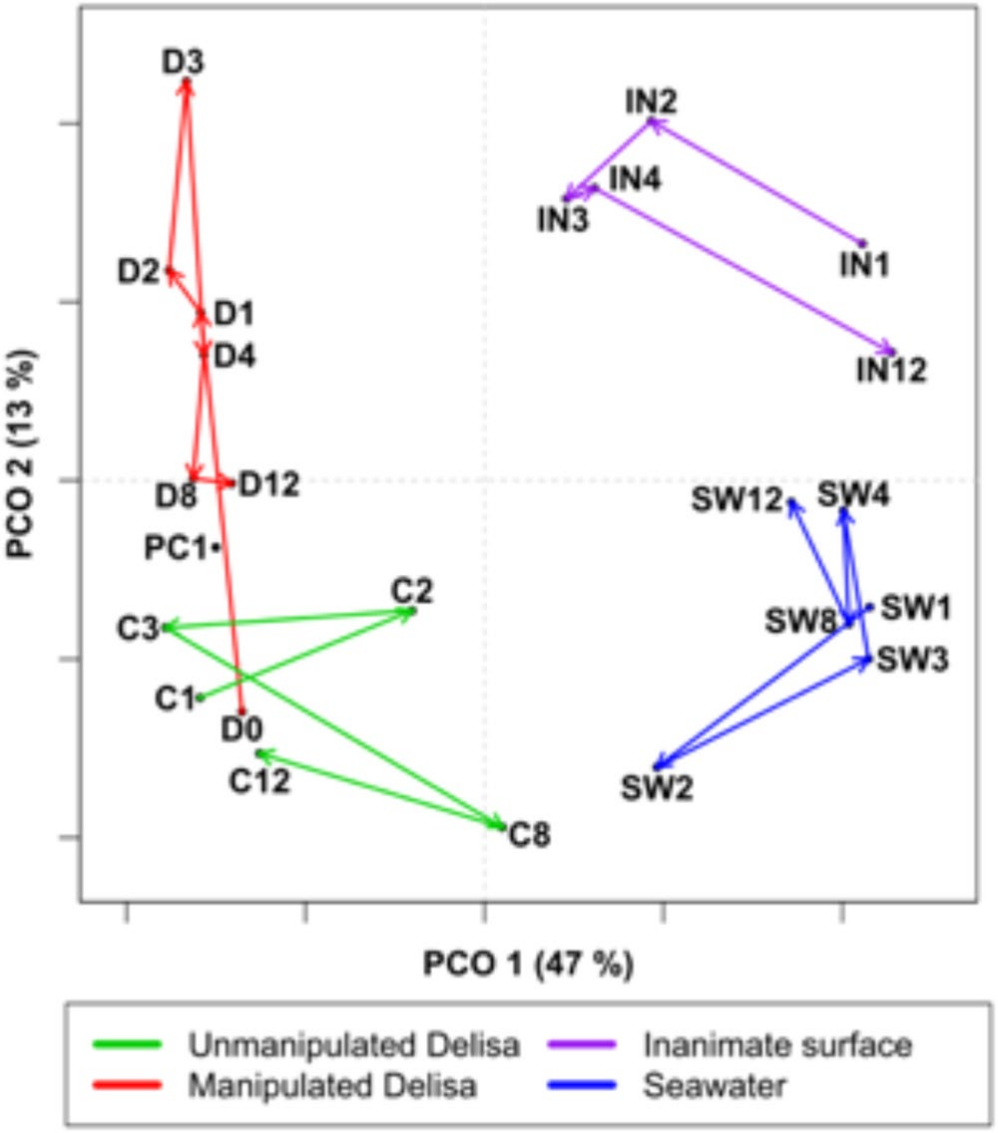
Principal component ordination (PCO) showing the average distances among microbiomes from *D. pulchra* whose microbiomes had been experimentally disturbed with antibiotic treatment (‘D’), procedural (‘PC’) and unmanipulated (‘C’) controls, and surrounding seawater (‘SW’) and inanimate surfaces (‘I’, deployed at the same time as the experimental algae) over time (0–12 d, as indicated by the number following the letter indicating treatment). Average distances between sample points were calculated from the distances observed from five independent experiments (see Methods) using the hierarchical sampling scheme with first level n = 5 and second level n = 1 replicates per comparison. Coloured arrows connect the sequential time points.

**Table 1:** Results of Mantel Tests examining cyclicity in changes of the composition of microbiomes according to DGGE banding patterns associated with the surface of *Delisea pulchra* over 12 days following an experimental disturbance.

Experiment #	Rho	P
1	0.505	0.038
2	0.545	0.039
3	0.214	0.231
4	0.476	0.058
5	0.075	0.372

Five separate experiments were analysed separately with n = 1–5 included in analyses, due to the use of hierarchical analysis of multiple DGGE gels (see Methods).

In all experiments, the average magnitude of community change in disturbed *D. pulchra* was much greater than for control algae or the surrounding environment (Fig. 1, Table 1). In order to generalise the trends observed, we averaged the pairwise distances between independent experiments and found that the patterns observed in the microbiomes of disturbed *D. pulchra* were consistently cyclic (Fig. 2; Table 2). This was unlike the microbiomes associated with unmanipulated control algae (Fig. 2; Table 2), which showed some level of temporal variation but no predictable pattern, and were overall much more stable than disturbed microbiomes (Fig. 2; Table 2). Similarly, background variation was observed in bacterial communities both in the seawater surrounding these experiments and on deployed inanimate surfaces (Fig. 2). Although these did not appear as cyclic, as for the manipulated *D. pulchra* (Fig. 2), they did conform statistically to the cyclic model (Table 2), however this appeared to be a result of overly influential points (indicative of poor model fit) rather than a truly cyclic pattern as observed in the disturbed *D. pulchra* microbiomes (Fig. S1). In the nMDS plots, no clear or consistent cyclic patterns can be observed in the microbial communities associated with inanimate surfaces.

**Figure 3:**
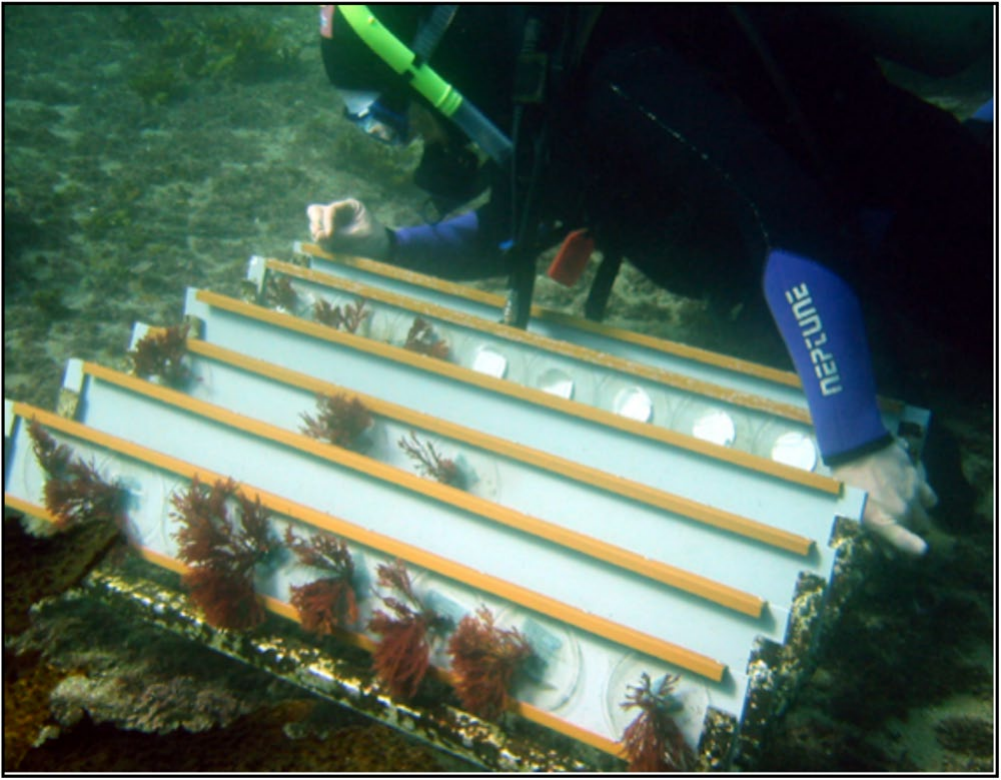
Photograph showing the custom-built stainless steel frame that was bolted onto the sea floor for the disturbance experiments, to which individual *D. pulchra* were attached for specific periods of time following disturbance of their microbiomes with antibiotic treatments.

**Table 2:** Results of Mantel Tests examining cyclicity in (a) experimentally disturbed *D. pulchra*, (b) unmanipulated *D. pulchra*, (c) surrounding seawater and (d) inanimate surfaces.

Treatment	*Rho*	*P*	# Permutations
Disturbed *D. pulchra*	0.41	0.049	999
Unmanipulated *D. pulchra*	−0.452	0.867	119
Seawater	0.591	0.006	719
Inanimate surfaces	0.652	0.05	119

Results from all five experiments were combined for these analysis, which used (a) 999, (b) 119, (c) 719 and (d) 119 permutations with n = 1–5 due to hierarchical analyses with multiple DGGE gels (see Methods).

### Culturing and phylogenetic identification of early and late successional bacteria from *D. pulchra*

To obtain isolates of early- and late-successional bacterial strains (‘ESS’ and ‘LSS’, respectively) for use in experiments designed to manipulate succession in *D. pulchra* (below), we collected healthy *D. pulchra* individuals, treated them with antibiotics and redeployed them in the field for either 1 or 14 days, then cultured the bacteria from their surfaces.

Fifteen isolates were cultured from *D. pulchra* individuals sampled at 1 d in the field, six of which were unique to 1 d and thus considered to be early successional strains (ESS). Another thirteen isolates were cultured from algal surfaces after 14 d in the field, four of which were unique to 14 d and these were considered later successional species (LSS). Of the 20 strains isolated, nine belonged to the *alpha-Proteobacteria*, four to the *gamma-Proteobacteria*, four to the *Actinobacteria*, two to the *Bacteroidetes* and one to the *Planctomyctes* clades (Table S1). Of these selected strains (six ESS and five LSS), only three ESS (ESS-06, ESS-23 and ESS-24) and one LSS (LSS-09) were distinctly pigmented (i.e. identifiable macroscopically) and thus suitable for use in manipulative assays (Table S1). Several residual (non-inoculated) strains were isolated from *D. pulchra* individuals sampled immediately after antibiotic treatments, all of which were still present on thalli sampled on 14 d, but in relatively low numbers.

### Experimentally manipulating bacterial succession within *D. pulchra*’s microbiome using inoculation experiments

We tested succession models of colonization in the context of within-microbiome interactions, by experimentally inoculating ecologically relevant ESS and LSS bacteria isolated from *D. pulchra* (above) onto *D. pulchra* individuals cultured in the laboratory to either produce (“furanone-plus”) or lack (“furanone-minus”) chemical defences (halogenated furanones).

**Figure 4:**
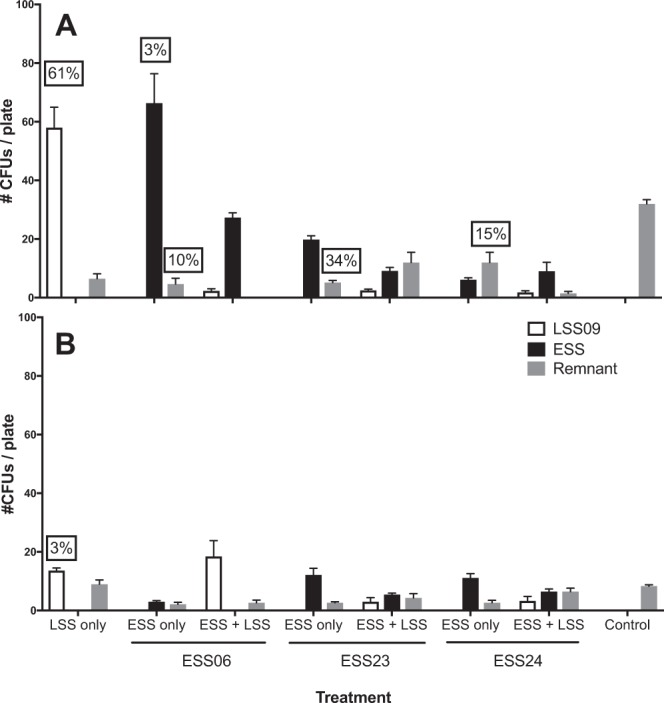
Average colony counts (±SE; bars; #CFUs) of ESS (ESS06, ESS23 and ESS24; black columns) and LSS (LSS09; white columns) bacterial strains cultured from the surfaces of *Delisea pulchra* sporelings (surface area ~30 mm^2^) cultured without (**A**) and with (**B**) chemical defences (halogenated furanones). Sporelings were exposed to either (i) ESS strains only, (ii) ESS then LSS strains, (iii) LSS strains only or (iv) no inoculation (‘control’). Residual bacteria were uninoculated strains present after the antimicrobial treatments (grey columns), with n = 9 for all treatments. Boxed numbers indicate the severity (average proportion of thallus area of replicates from each treatment) of thallus bleaching resulting from inoculation x chemical defences treatments (absence of box indicates no bleaching in that treatment).

On furanone-minus *D. pulchra*, the three ESS isolates tested (ESS06 [*Actinobacteria*], ESS23 [*alpha-Proteobacteria*] and ESS24 [*alpha-Proteobacteria*]) all inhibited colonization of LSS09 [*alpha-Proteobacteria*]) however LSS09 was able to colonize furanone-minus thalli in the absence of ESS isolates Fig. 4A; Table 3). Colonization of ESS06 was significantly reduced on furanone-plus individuals, whereas ESS23 and ESS24 were able to colonize chemically defended thalli (although densities of ESS23 were significantly lower than on furanone-minus thalli, whereas densities of ESS24 were not significantly different among algae with and without chemical defences; Fig. 4A, Table 3). Densities of LSS09 were significantly reduced on furanone-plus thalli and this reduction was similar to that observed in the presence and absence of previously inoculated ESS strains (Fig. 4B, Table 3). Two out of three ESS isolates (ESS06 and ESS23) and LSS09 colonized furanone-minus thalli more effectively in the absence of other inoculated strains, whereas densities of ESS24 were not significantly different among inoculation regimes (Fig. 4B, Table 3).

**Table 3:** Analyses of variance (ANOVAs) comparing the abundance of colony forming units (CFUs) of ESS and LSS bacterial strains inoculated in various combinations onto *D. pulchra* sporelings lacking (‘furanone-minus’) or producing (‘furanone-plus’) chemical defences.

Source	*df*	*MS*	*F*	*P*	*df*	*MS*	*F*	*P*
	(a) ESS06 in the presence/absence of LSS09				(b) LSS09 in the presence/absence of ESS06			
Furanone (F)	1	56.30	127.31	0.06	1	0.17	0.01	0.93
Inoculation (I)	1	7.17	142.94	<0.01	1	12.57	51.16	<0.01
F × I	1	0.44	8.81	0.01	1	14.54	59.17	<0.01
Error	20	0.05			20	0.25		
	**(c) ESS23 in the presence/absence of LSS09**				**(d) LSS09 in the presence/absence of ESS23**			
Furanone (F)	1	1.36	83.60	0.07	1	2.90	1.10	0.49
Inoculation (I)	1	2.86	34.12	<0.01	1	28.00	177.85	<0.01
F × I	1	0.02	0.19	0.66	1	2.64	16.74	<0.01
Error	20	0.08			20	0.16		
	**(e) ESS24 in the presence/absence of LSS09**				**(f) LSS09 in the presence/absence of ESS24**			
Furanone (F)	1	9.38	0.11	0.80	1	1.72	3.45	0.31
Inoculation (I)	1	5.04	0.15	0.71	1	2.49	3.06	0.02
F × I	1	84.38	2.46	0.13	1	0.50	1.21	0.28
Error	20	34.36			20	0.41		

Inoculation combinations are (a) ESS06 in the presence/absence of LSS09, (b) LSS09 in the presence/absence of ESS06, (c) ESS23 in the presence/absence of LSS09, (d) LSS09 in the presence/absence of ESS23, (e) ESS24 in the presence/absence of LSS09 and (f) LSS09 in the presence/absence of ESS24, with n = 9 in all experiments.

Furanone-minus thalli inoculated with LSS09 in the absence of ESS isolates, bleached significantly more than controls, with an average of 60% of thallus-area of individuals in this treatment bleaching (Fig. 4, Table 4). As with the patterns of colonization, the presence of ESS bacteria appeared to protect furanone-minus thalli from bleaching when they were also inoculated with LSS-09. In these cases bleaching levels were significantly reduced, with some ESS strains preventing bleaching more effectively than others (e.g. bleaching was reduced to <10% of the thallus by ESS-06, approximately 15% by ESS23, and ~ 35% by ESS24; Fig. 4, Table 4). Furanone-minus thalli inoculated with ESS06, ESS23 or ESS24 only (i.e. no LSS09) did not bleach significantly more than controls (Fig. 4A, Table 4). Furanone-plus algae did not bleach significantly more than controls, regardless of the inoculation regime to which they were allocated (Fig. 4B, Table 4).

**Table 4:** Analysis of variance (ANOVA) comparing the proportion of chemically defended (‘furanone-plus’) and undefended (‘furanone-minus’) *D. pulchra* thalli (% area) that bleached among inoculation treatments: (a) ESS06 +/− LSS09, (b) ESS23 +/− LSS09 and (c) ESS24 +/− LSS09, with n = 9 in all experiments.

Source	(s) ESS06 +/− LSS09				(b) ESS23 +/− LSS09				(c) ESS24 +/− LSS09			
	*df*	*MS*	*F*	*P*	*df*	*MS*	*F*	*P*	*df*	*MS*	*F*	*P*
Furanone (F)	1	0.112	193.133	<0.001	1	0.345	633.260	<0.001	1	0.219	396.374	<0.001
Inoculation (I)	3	0.151	260.046	<0.001	3	0.158	282.807	<0.001	3	0.154	277.577	<0.001
F × I	3	0.001	269.398	<0.001	3	0.135	240.560	<0.001	3	0.127	229.275	<0.001
Error	16				16	0.001			16	0.001		

The numbers of residual bacteria were consistently low across all assays. Although residual strains had two- to three-fold higher densities on furanone-minus than furanone-plus thalli in all assays, no differences were observed in bleaching between furanone-minus and furanone-plus controls, so residual strains were not considered to confound the experimental results (Fig. 4).

## Discussion

In one of the first studies to experimentally investigate the impacts of within-microbiome interactions on host health, we found that microbiomes associated with *Delisea pulchra* showed strong, directional patterns of community change following a major disturbance. After only 12 days, experimentally disturbed microbiomes returned to a pre-disturbance state, which in most cases was statistically indistinguishable from unmanipulated, ‘native’ microbiomes. In manipulative experiments designed to interfere with this succession process, we found that several early-colonizing strains of bacteria protected the algal host from putative pathogens that are more likely to colonize during later successional stages and cause disease. *D. pulchra*’s chemical defences – halogenated furanones – appear to exert an important selective force on this alga’s microbiome, both with respect to recovery following a disturbance and *via* inhibition of potential pathogens. Thus, when the production of these chemical defences is compromised, such as during times of e.g. increased water temperatures^35^, the protective, inhibitory properties of early bacterial colonizers against later colonizing pathogens may become an important factor for the health of the host.

### Resistance, resilience and persistence in *D. pulchra*’s microbiome

The patterns of change in *D. pulchra*’s microbiome following the experimental disturbance were directional (cyclic), demonstrating predictable successional processes were taking place. This cyclic pattern of recovery was consistently observed in four separate experiments conducted over two years, with some variability (e.g. in the third experiment, a clear cyclic pattern was observed but the rate of succession between sampling days was dissimilar to other experiments). The shifts observed in microbiomes on experimentally manipulated *D. pulchra* were greater in magnitude than those observed in seawater communities and biofilms on inanimate surfaces over the same time period. Such stability has been observed in epiphytic bacterial communities associated with another macroalga - the green alga *Caulerpa taxifolia*, whose microbiomes are so stable they can be used to trace the origins of the alga itself ^38^.

Interestingly, our results and those from *C. taxifolia*^38^ are contrary to a systematic review conducted by Allison & Martiny^39^, who found that the vast majority of bacterial communities associated with (predominantly terrestrial) plants were not resistant to various environmental disturbances (including temperature, CO_2_, nutrient and changes in carbon sources) and often did not recover, even after years, potentially reflecting differences in holobiont dynamics between marine and terrestrial environments.

One factor which may facilitate such stability is the diversity of *D. pulchra*’s microbiome. Diversity has been linked to stability and resilience in soil microbial communities, which can withstand extreme perturbations (e.g. from pollution and cultivation) more effectively when bacterial diversity is high^40^. We have previously demonstrated that *D. pulchra* has a diverse microbiome compared to other macroalgae, using comparable methodology (>62 OTUs in the tip region alone^41^; compared to 28 for *Ulva* spp. and 18 and 14 for *Sargassum* spp. and *Porphyra* spp. respectively). In the context of environmental change, a more diverse microbial community might provide insurance against perturbation, with continued stability in community structure through time^42,43^, although there is some debate about the generality of this for microbial communities^44,45^.

### Chemical defences and priority effects

Although initial colonization following a disturbance is often a stochastic process^46^, the presence of biologically active compounds (halogenated furanones) in algal tissues may have influenced which bacterial strains were able to persist, thereby asserting a host-mediated influence on the composition of early successional communities (e.g. ESS-06 was unable to colonize chemically-defended sporelings). This suggests that succession within the microbiome of *D. pulchra* may be deterministic whenever ecologically relevant levels of halogenated furanones are available within the host tissues. Furthermore, prior to random selection of replicates for hierarchical analysis of DGGE gel banding patterns, we compared all replicates from within each treatment on single gels (data not shown). We observed very low levels of between-replicate variability at all time points in all experiments, indicating a high degree of uniformity among individuals at corresponding times in the succession process. Given the high diversity of epiphytic bacteria associated with *D. pulchra*, it is surprising that community composition remained so uniform across replicates at corresponding times and that succession patterns were indeed reproducible.

Recent studies have highlighted the importance of priority effects, where the starting community has long-term implications for microbial community structure^17,26^, so potentially halogenated furanones, which are maintained at the algal surface boundary layer via vesicle cells^30^ could strongly influence which OTUs can settle first, with long- term effects on the resulting community. Subsequently, this macroalga’s production of halogenated furanones may influence the composition of its microbiome, returning it to equilibrium following a disturbance, to the benefit of the host’s health and performance.

When space becomes available after a disturbance, colonists may come from either the environment, or regrow from remnant, residual colonies that survived the disturbance^47^. In this case, the source of bacterial colonists onto *D. pulchra*’s surface is not obvious because neither the communities in the surrounding seawater, nor those that developed on inanimate surfaces attached adjacent to the experimental algae had any overlap with *D. pulchra*’s microbiomes, in terms of composition. Potentially, *D. pulchra* selects (*via* the production of halogenated furanones and other chemical, physical and phenological traits), relatively rare species that may exist in the water column in low abundance (and thus remain undetectable by DNA fingerprinting methods). These may impart competitive traits suited to the specific environment of the host algal surface, where they flourish and influence the resulting composition of the developing microbiome.

Our observation of complete recovery of severely disturbed microbiomes after only 12 days *in situ* is remarkable, given the diverse nature of the community and highlights the utility of using bacterial populations to test ecological theories. Although generation times vary greatly among bacteria (minutes to hours to days^48^), as a group, they are dramatically shorter than most multicellular eukaryotes (weeks to years to decades). In the present study, we (conservatively) observed dozens of generations of bacterial communities. Following a similar number of generations of eukaryotes used in classical successional studies such as trees, grasses or corals during their recovery from a disturbance back to equilibrium would require decades if not centuries. Indeed, other micro-fouling (both prokaryote and eukaryote) communities provide further support for usefulness in assessing long-held ecological theories, for which empirical evidence may be lacking, varied, or inconclusive, over many generations. For example, one study of microbiomes on inanimate surfaces placed in a lake, reported rapid change in biofilm community structure in the initial 3–4 d, with stability observed after only 30 d *in situ*^49^.

### Interactions within the microbiome of *D. pulchra* and impacts on the host

By experimentally manipulating both the production of algal chemical defences and the presence/absence of early- and late-successional bacteria, we learned that both host-mediated effects and interactions between members of the microbiome, can strongly affect succession and have major implications for host health. When algal chemical defences were left intact, colonization by the putative pathogen LSS09 was largely inhibited and algal bleaching minimal. When algal chemical defences were experimentally inhibited, LSS09 was able to colonize *D. pulchra* and when it did so, caused widespread bleaching. However, the presence of ESS strains significantly reduced both colonization by LSS09 and subsequent bleaching. A similar phenomenon was observed in laboratory trials using maize roots, where a simplified bacterial community prevented the growth of a pathogenic fungus^16^.

Halogenated furanones inhibit bacterial cell-cell communication and the phenotypes they regulate by interfering with quorum sensing in bacteria that produce acylated homoserine lactones (AHLs^31,50^). Recent related work has identified LSS09 as a dominant member of microbiomes associated with both bleached and healthy *D. pulchra* collected from the field^51–53^. Additionally, several putative virulence factors and a quorum-sensing dependent transcriptional regulator have also been identified^51^, which, in combination with our results, suggests that *D. pulchra*’s chemical defences may interfere with both (i) attachment and settlement and (ii) infection and bleaching. Fernandes *et al*.^51^ proposed that LSS09 may be a member of *D. pulchra*’s native microbiome (due to its detection within the microbiomes of healthy adults from their natural environment) that can become pathogenic opportunistically, under certain conditions. Our work suggests that both a reduction of tissue concentrations of halogenated furanones and a disturbance within *D. pulchra*’s microbiomes (e.g. *via* grazing or scouring) may facilitate LSS09 switching to a pathogenic lifestyle, leading to algal bleaching.

Bleaching leads to dramatic reductions in fecundity, reduced growth and enhanced susceptibility to grazing, which all have deleterious impacts on affected individuals^37^. There is considerable spatial and temporal variability in the concentration of halogenated furanones in *D. pulchra* individuals^35,54^ and under certain conditions (e.g. high water temperatures^35^) concentrations can be very low. This is concerning given that climate conditions are predicted to become more extreme, potentially creating scenarios in which *D. pulchra’s* chemical defence will be compromised in the future. Under such circumstances, *D. pulchra*’s microbiome may be its salvation: The protective role early-successional species appear to play may become more important for host health if algal chemical defences are reduced or depleted entirely. In our experiments, all three ESS strains prevented LSS09 colonization and significantly reduced the frequency and severity of the bleaching it caused, compared to colonization and disease that occurred in their absence. These observations provide evidence for the inhibition model of succession within *D. pulchra*’s microbiome.

Although compelling, an important caveat to this study is that only three ESS and one LSS strains were used – it is possible that other ESS strains may not have inhibitory effects against this or other LSS strains. However, our observations do suggest that early colonizers in *D. pulchra*’s microbiome may follow the classic pattern of inhibition as observed in many other systems^55^ and may be playing an important protective role for this alga when its own chemical defenses are depleted. Another limitation of our study is the use of the DNA fingerprinting methodology DGGE, which has been increasingly replaced with newer, higher throughput, next generation sequencing technology to explore the diversity and distribution of microbiomes from diverse habitats on a global scale. Whilst the use of DGGE is constraining in some respects, a major benefit of this technique is that you can obtain the full-length 500 bp sequence, providing better coverage for analysis of taxonomic patterns than shorter reads from, for example, MiSeq sequencing (discussed in ref.^56^). Furthermore, for the *D. pulchra* holobiont specifically, we now know that patterns of composition of its microbiome based upon DNA fingerprinting techniques such as DGGE as described here, and sequencing from clone libraries (e.g.^35,51^) are similar to patterns obtained using deep sequencing of the 16S rRNA gene^53^, so we are confident in the validity and reproducibility of our results from DGGE analysis as presented. Our results suggest that the application of ecological theory, such as succession theory, is a useful approach for investigations into interactions between microorganisms within microbiomes and their consequences for host health.

## Methods

### Study location and organism

*Delisea pulchra* (Greville) Montagne (Bonnemaisonales: Rhodophyta) is a red macroalga, common in subtidal habitats between one and fifteen meters depth in coastal habitats around south-eastern Australia. Algal collection and ‘disturbance experiments’ took place on rocky reefs at depths of 2–4 m at Bare Island in Botany Bay (151°13′50″E, 33°59′32″S) near Sydney, Australia. The reefs at this site are dominated by macroalgae, most commonly the foliose species *Ecklonia radiata* (C. Agardh) J Agardh (Laminariales: Heterokontophyta), *Sargassum linearifolium* (Turner) C. Agardh (Fucales: Heterokontophyta) and the turfing corallines *Corallina officinalis* (Linneaus) (Corallinales: Rhodophyta) and *Amphiroa anceps* (Lamark) Decaisne (Corallinales: Rhodophyta). During the period of this study, *D. pulchra* was common and healthy at these depths.

### Responses of *D. pulchra*’s microbiome to experimental disturbance over time

To investigate how the microbiome associated with *Delisea pulchra* changed following a disturbance, we experimentally disturbed alga-associated biofilms then monitored their recovery over time. We did this by removing thalli from the benthos, exposing them to a combination of antibiotics then replanting them back onto their reef of origin. Using DNA fingerprinting techniques, we then monitored how the composition of the microbiome changed over time, compared to various controls and the surrounding environment, over the same time period. This experiment was repeated five times: twice in 2002 and three times in 2004.

Mature *Delisea pulchra* individuals (n = 30) were inspected *in situ* using SCUBA and if assessed as ‘healthy’ (no visible signs of bleaching, as per^35^), collected and transported in seawater (SW) collected onsite to the laboratory. To experimentally disturb the surface-associated microbiome, each alga was soaked in sterile filtered seawater (SFSW) with 10% iodine solution for 2 min, then placed in an individual 2 l beaker containing SFSW with added antibiotics (20 mg/l streptomycin, 10 mg/l kanamycin and 10 mg/l penicillin G) and aerated for 24 h at a constant temperature of 19–20 °C to approximate field conditions. Prior to these experiments, pilot studies confirmed that this combination of antibiotic treatments significantly reduced biofilms on treated algal surfaces while leaving algal cells intact. Procedural controls were collected at the same time as antibiotic treated algae and handled in the same way, except they were soaked in SFSW that contained no iodine or antibiotic solutions.

Following antimicrobial treatments, each alga was rinsed and then ‘replanted’ back onto the reef from where they had been collected the previous day. To do this, we used cotton thread to stitch the holdfast of each alga into plastic mesh that had previously been glued to the inside of individual, plastic Petri dishes (9.0 cm diameter). The Petri dishes with experimental algae attached were transported back to the field in SFSW and then using SCUBA, slotted into a custom-built stainless-steel frame that had been bolted to the benthos adjacent to the collection site for these experiments (Fig. 3). This frame facilitated effective reattachment of the algae back into the reef, without the need for any further manipulations. To compare changes in *D. pulchra*’s microbiome to changes in other microbial communities from the same environment over the same time period, we also deployed experimental ‘inanimate surfaces’, which were sterile cellulose ester 0.45 μm filter membranes (Pall Gelman Laboratory), attached to Petri dishes slotted into the same frame as experimental *D. pulchra* thalli, and also collected seawater samples (1 l) to investigate changes in the planktonic microbial community surrounding the experimental rig. Finally, on each collection date, we also destructively sampled unmanipulated *D. pulchra* individuals (n = 5) from the substratum to compare ‘background’ changes in algal microbiomes to those resulting from our manipulations. These treatments (antibiotic, procedural control and unmanipulated control) were chosen to assess how an experimental disturbance (via exposure to antibiotics) would affect the microbiome and any impacts this might have on the host.

Samples (n = 5) were collected 1, 2, 3, 4 and 12 d after deployment in the 2002 experiments and 1, 2, 3, 4, 8 and 12 d after deployment in the 2004 experiments. Each individual alga or inanimate plate was enclosed within a sterile press-seal plastic bag *in situ* and water samples were collected directly adjacent to the experimental frame using sterile 1 l Nalgene bottles. Following collection, each alga and inanimate surface was rinsed twice in SFSW to eliminate unattached bacteria prior to transport (on ice) to the laboratory where they were rinsed three more times in SFSW before processing. The tips (top 2–3 cm) of all algal thalli (disturbed, procedural and unmanipulated controls) were aseptically removed for further processing. SW samples were passed through 0.2 μm filter paper to concentrate bacterial cells. Algal tissue and filter membranes (both water filtrate samples and experimental inanimate surfaces) were frozen at −20 °C then freeze-dried in preparation for DNA extraction.

### DNA extraction, PCR amplification and denaturing gradient gel electrophoresis (DGGE) and sequencing of excised bands

DNA of microbiomes was extracted from freeze-dried *D. pulchra* samples (30 mg) and filter membranes (both the experimental inanimate surfaces and SW filtrates) using the ‘bead-beating’ method as per^57^. PCR amplification of bacterial 16S rDNA was conducted using the primers 341F-GC and 907RC (Schäfer and Muyzer, 2001) with the cycling regime: 94 °C (3 min), hot start at 80 °C, 25 cycles of 94 °C (30 sec), 57 °C (1 min 30 s), 72 °C (1 min), with a final extension at 72 °C (10 min). Denaturing gradient gel electrophoresis (DGGE) is a DNA fingerprinting method that is useful to compare microbiome community structure among environmental samples such as seaweed surfaces^58^ and we used this method (using a Bio-Rad DCode system) to compare microbiomes associated with algal and inanimate surfaces and surrounding seawater from these experiments. A denaturing gradient of 35–55% was used in a 6% polyacrylamide gel. Gels were run at 75 V for 16 h at 60 °C, after which they were stained for 20 min in SYBR Gold (Molecular Probes) and photographed with a Bio-Rad Gel-Doc 2000 Imaging System. Resulting DGGE band positions were marked using the Bio-Rad QuantityOne software (Version 4.0.3) and their presence/absence recorded in a matrix.

Bands of interest (n = 20) were excised from the gels for sequencing using a sterile scalpel blade and eluted in 20 μl molecular grade water overnight at 4 °C. The eluent was used as template DNA for PCR amplification with the same 16S rDNA primers used above. PCR products were cleaned using the QIAquick PCR Purification Kit (Qiagen) and sequenced using the 907RC primer and the BigDye Terminator RR Mix. The resulting product was sequenced, with a reaction involving one cycle of 94 °C for 3 min, the 60 cycles of 94 °C for 10 sec, 50 °C for 5 sec and 60 °C for 4 min. Sequencing products were precipitated in 50 μl ethanol and 2 μl sodium acetate (pH 5.2) for 20 min at room temperature. DNA was collected by centrifugation (14,000 rpm, 30 min, 4 °C) and the pellet washed, recentrifuged and washed again using 70% ethanol.

One of the constraints of using DGGE is that samples cannot easily be compared among gels, thereby reducing the amount of replication possible. The banding pattern of one DGGE gel is unique to that gel and has no equivalent on other gels because manual pouring of the gels results in slight differences in the gradient. Comparison of presence/absence of bands across the whole data set is therefore not possible. Hierarchical analysis was adopted to overcome this limitation, whereby replicate samples from each time point (n = 5) were initially run together on gels (with up to 20 lanes in this study) and evaluated for similarity/differences. If replicates were 80% or more similar, representatives of these samples were randomly selected and run concurrently on the one gel, thus giving a representation of all samples on the one gel and allowing direct comparison among treatments and sample types (see^59^ for similar analyses).

A benefit of using DGGE is that each band contains the DNA of a particular organism such that bands of interest can be excised and the DNA within sequenced. We were able to identify bacterial sequences using this method and excised bands were sequenced at the Ramaciotti Centre, UNSW. Consensus sequences were assembled using SeqEd Version 1.0.3 (Genetics Computer Group, Applied Biosystems). Approximate phylogenetic affiliations were determined by comparing the excised sequences with those in publicly available databases using BLAST (http://www.ncbi.nlm.nih.gov/BLAST).

### Experimentally manipulating chemical defences and bacterial succession within *D. pulchra’s* microbiome

Based on the results of the disturbance experiments (above), we became interested in differences between bacterial strains that colonized manipulated thalli in the first few days following the disturbance (early successional strains; ESS) and those that colonized later on (later successional strains; LSS). In order to develop a collection of the culturable members of both the ESS and LSS bacterial communities to use in experiments, we collected healthy, adult *D. pulchra* (n = 20), disturbed their microbiome (as described above), replanted them back into the field (as described above), then sampled the culturable bacteria from experimental thalli over time (1 and 14 d) using the spread plate method. This involved the aseptic removal of algal tips, which were then vortexed in 20 ml SFSW using a desktop vortex at maximum speed for 20 min (as per^41^). Tips from each individual thallus (n = 3) were pooled and three replicates plated for each serial dilution. The algal sections were removed from the solution and the remaining bacterial suspension was centrifuged at 2500 rpm for 5 min to concentrate the cells. After removing the supernatant, the cells were resuspended in 20 ml sterile seawater and inoculated onto Marine Broth (Difco) (1.5% agar) spread plates in serial dilution. Colonies were picked according to distinctive colony morphology and re-streaked until pure isolates were obtained.

Isolates were categorized according to succession status as early (ESS; 1 d) or later succession species (LSS; 14 d). Any isolates that were cultured from more than one time-point were recorded separately according to when they were isolated. If the phylogenetic identity was the same for isolates cultured at different time-points but their colony morphology differed, they were categorized separately. Many of the isolates were non-pigmented or had stopped producing pigment in culture and were therefore more difficult to identify by colony morphology alone. The differences in colony morphology used to identify the non-pigmented isolates (e.g. surface texture) were often subtle, rendering these isolates less amenable to the experimental protocol used here. Thus, four pigmented isolates (three ESS (ESS06, ESS23 and ESS24) and one LSS (LSS09) were chosen to be used in inoculation experiments because they were identified as belonging to solely one time-point, had discreet colony morphology and were previously identified in clone library analyses^41^.

Once the isolates to be used in manipulative experiments had been selected, we assessed the lowest concentration of cells that would allow colonization *in vitro*. This involved inoculating replicate furanone +/− *D. pulchra* sporelings with a dilution series (10^2^, 10^3^, 10^4^, 10^5^, 10^6^, 10^7^ and 10^8^ cells/ml) of each isolate. The optimal cell concentration was found to be 10^6^ cells/ml and this concentration was used in all subsequent experiments.

To identify our isolates, DNA templates were extracted directly from pure colonies by lysing cells in 20 μl molecular grade water at 98 °C for 10 min. The samples were centrifuged (13000 rpm for 2 min) to form a pellet and the supernatants used as templates. 16S rDNA gene fragments were amplified using the bacterial primers 27 F and 1492 R (as above; S1). Amplifications were performed in a Hybaid PCR Express thermocycler under the following conditions: 3 min denaturing at 94 °C; hot-start at 80 °C; 25 cycles of 1 min denaturing at 94 °C, 1 min annealing at 55 °C, 2 min extension at 72 °C; final extension of 10 min at 72 °C. Bacterial isolates were identified using 16S rRNA gene sequences. PCR products were purified using the QIAquick PCR purification kit (Qiagen) and sequenced using 27 F, 507 R and 1492 R primers. Consensus sequences were assembled using SeqEd Version 1.0.3 (Genetics Computer Group, Applied Biosystems). Sequencing analysis of bacterial isolates was conducted on 16S rDNA from the PCR following the protocol outlined above (S1). Closest phylogenetic relatives were found using the publicly available databases, Basic Local Alignment Search Tool (BLAST) (http://www.ncbi.nlm.nih.gov/BLAST). Sequences were aligned using the FastAlign function within the ARB package (http//www.arb-home.de) and alignments were refined manually. Phylogenetic trees were constructed using evolutionary distance analyses on near full-length 16S rDNA sequences (>1300 nt). Bootstrap analysis (1000 resamplings) tested the robustness of tree topology using parsimony methods in ARB. Maximum likelihood trees were also constructed in ARB using the fast DNAml method to validate tree topology. Previously sequenced clones^41^ were included in the tree construction and DGGE-derived sequences were subsequently added (using the ARB Parsimony Interactive tool) without changing tree topology^41^.

To obtain approximate phylogenetic affiliations for the sequenced isolates, each sequence was subjected to BLAST analysis against the GenBank database (www.ncbi.nlm.nih.gov/BLAST/). Sequences from this study, and their close relatives derived from GenBank, were aligned in the ARB package^60^ using the FastAligner function. All alignments were then manually refined. Maximum likelihood-based phylogenetic analysis was conducted in ARB (AxML) using near full-length (>1300 bp) sequences only, and the robustness of tree topologies tested by parsimony-based bootstrap analysis (1000 resamplings). Sequence data have been submitted to the GenBank database under accession numbers MH057241-MH057259.

The isolates we obtained were categorized according to succession status as either early (ESS; 1 d) or late succession species (LSS; 14 d; Table S1) and divided into groups of distinctly pigmented bacteria (S3). Isolates were grown in pure culture for DNA extraction and sequencing (Ramaciotti Centre, UNSW) then identified using phylogenetic analyses. Isolates that were observed in only one succession status category (ESS or LSS), with distinct colony morphology and which had been previously identified in clone library analyses^41^ were selected and used in inoculation assays at a concentration of 10^6^ cells/ml, which we found to be the lowest concentration at which colonization would occur *in vitro*.

ESS and LSS bacterial strains were inoculated onto *D. pulchra* sporelings grown in the laboratory. We also manipulated chemical defences by culturing sporelings either containing (‘furanone plus’) or lacking (‘furanone minus’) halogenated furanones - chemical defences that can interfere with bacterial cell-cell signaling systems (quorum sensing;^31,50^) and therefore inhibit the settlement of many bacterial strains^61^, using established methods e.g.^35,36^. Using this method, furanone plus/minus sporelings are morphologically and demographically similar, other than their concentrations of secondary metabolites^36^. Prior to experimentation, furanone plus/minus *D. pulchra* sporelings were treated with antibiotics (as described above) then transferred to 2 ml sterile, artificial seawater (ASW; containing bromide (Br+) for furanone plus algae and lacking bromide (Br−) for furanone minus algae).

Chemically defended and undefended thalli were exposed to bacterial inocula (ESS or LSS; 10^6^ cells per ml) for an initial 3 d, after which they were rinsed three times in sterile ASW (Br+/−) then re-immersed in fresh inocula (ESS or LSS; 10^6^ cells per ml) for another 3 d. Three pigmented ESS (ESS06, ESS23 and ESS24) were each assayed against LSS-09 in four inoculation regimes: (1) ESS for the first 3 d, followed by fresh ESS inoculum for another 3 d, (2) LSS for the first 3 d, followed by fresh LSS inoculum for another 3 d, (3) ESS for the first 3 d, followed by LSS inoculum for the final 3 d and (4) control, treatment, where ASW was used instead of bacterial cultures. Control treatments were included to capture residual bacteria that survived the antibiotic treatments and detect any experimental handling effects on the *D. pulchra* holobiont.

At the end of the experiment (on the sixth day), colonization success, as well as impacts on the host % thallus bleaching, as per^35,62^ were quantified. To quantify bacterial colonization onto algal surfaces, each alga was again rinsed three times in ASW (Br+/−) then vortexed for 20 min in 200 μl ASW (Br+/−). Dilutions (10^−2^) of this seawater suspension were inoculated onto Marine Agar (Difco) (1.5% agar) in 10 μl aliquots using the spread-plate method and incubated at room temperature for 5 d. The resulting bacterial colonies were counted and identified based on colony morphology. The proportion of each individual thallus that was bleached was quantified visually using a dissection microscope, then recorded and compared among treatments.

### Statistical analyses

One of the constraints of using DGGE is that samples cannot be compared between gels, thereby reducing the amount of replication possible. To overcome this limitation replicates run on separate gels (with up to 20 lanes) and evaluated statistically for similarity/differences using PRIMER^63^. If found to be not significantly different, representatives of these samples can be randomly selected and run concurrently on the one gel, thus giving a representation of all the samples that can be compared directly on the one gel.

For each of the five, independent ‘disturbance experiments’, DGGE band positions were marked using the Bio-Rad QuantityOne software (Version 4.0.3), recorded manually in a presence/absence matrix and then compared among treatments (disturbed *D. pulchra*, unmanipulated *D. pulchra*, inanimate surfaces and seawater) using a Bray-Curtis similarity matrix (which for presence/absence data such as these, generalises to Sorrenson). Principal correlation ordination (PCO) was used to visualise distances between samples and to assess whether there was a cyclic pattern in the manipulated *D. pulchra*. For an objective measure of cyclic patterns, we used Mantel tests for correlations of distance matrices^64^; to compare rank distances between community profiles and that of a ‘model matrix’ containing rank distances pertaining to a cyclic pattern^63,65^. The ‘model matrix’ was based on the distances from the first experiment and ranked communities with lower values between shorter time periods (days), higher values between longer time periods with the largest ranks assigned between 4 d and either 0 d or 12 d - the other being assigned the smallest rank.

To aggregate the data among the experiments, we averaged the distances between all time point pairs (e.g. the average of distances between 0 d and 1 d for manipulated *D. pulchra* observed among experiments) and generated a new ‘average’ distance matrix. PCO and Mantel tests were again used to visualize the distances between time points and among treatments, and to determine the presence of cyclic patterns, respectively, but on the average distances between microbiome communities associated with different surfaces/seawater and over time rather than within each experiment.

Colonization of inoculated bacteria during the inoculation experiments was analyzed by a series of two-factor analyses of variance (ANOVAs) where the treatments were chemical defences (present/absent) and inoculation (ESS/ESS, LSS/LSS or ESS/LSS). Colony counts for ESS and LSS were each analyzed separately (across the bromine plus/minus treatments) to establish the effects each had on the other. That is, (i) ESS colony counts were analyzed for inoculation treatments of ESS only and compared to colony counts resulting from inoculations of ESS followed by LSS and; (ii) LSS colony counts were analyzed for inoculation treatments of LSS only and compared to colony counts resulting from inoculations of LSS that followed exposure of the algae to ESS. This approach enabled the effects of ESS/LSS inoculation combinations to be assessed independently for both the early colonizers (ESS) and the later colonizers (LSS). The proportion of thalli that bleached in the inoculation experiments was analyzed using the same two-factor ANOVAs described above.

All analyses were conducted using the statistical platform ‘R’ (http://www.R-project.org) using the packages ‘stats’ and ‘vegan’^66^.

## Supplementary information

Supplementary Information, Longford et al.

## Data Availability

Raw data are available at https://figshare.com/s/65ef603011b41d7fddc7.
