# Supplementary material for: Interactions within the microbiome alter microbial interactions with host chemical defences and affect disease in a marine holobiont
Source: Sci Rep. 2019 Feb 4;9:1363. doi: 10.1038/s41598-018-37062-z (PMC6361982; doi:10.1038/s41598-018-37062-z)
Supplement: Supplementary file 1 — Supplementary Information, Longford et al. [file 41598_2018_37062_MOESM1_ESM.pdf]

Interactions within the microbiome alter microbial interactions with host chemical defences and affect disease in a marine holobiont

Sharon R. Longford[1,2], Alexandra H. Campbell[1,3,4*], Shaun Nielsen[1], Rebecca J. Case[5], Staffan Kjelleberg[1,2,6] & Peter D. Steinberg[1,2,3]

1. Centre for Marine Bio-Innovation, and School of Biological, Earth and Environmental Sciences, University of New South Wales, Sydney NSW 2052, Australia

2. Singapore Centre for Environmental Life Sciences Engineering, 60 Nanyang Drive, SBS-01N-27 Singapore 637551

3. Sydney Institute of Marine Science, Chowder Bay Road, Mosman, NSW, 2088, Australia

4. GeneCology Research Centre, University of the Sunshine Coast, Sippy Downs, QLD, 4556, Australia

5. Department of Biological Sciences, Biological Sciences Centre, University of Alberta, Edmonton, Alberta, Canada T6G 2E9

6. School of Biological Sciences, Nanyang Technological University, 60 Nanyang Drive, Singapore 637551

* *Corresponding author*: Alexandra H. Campbell; Current address: L3, Building T, University of the Sunshine Coast, 90 Sippy Downs road, Sippy Downs, QLD 4556

Keywords: macroalgae, bacteria, experimental ecology, chemical defenses, DGGE

Table S1. Isolates (ESS-xx and LSS-xx) collected from D. pulchra thalli, after 24 hours ('Day 1') and 14 days ('Day 14') of redeployment in the field at Bare Island in Botany Bay (151°13'50'' E, 33°59'32'' S) near Sydney, Australia. Phylogenetic affiliation, closest taxon (including from *D. pulchra*'s clone library, indicated with "DPCxxx"; Longford et al., 2007) and percentage similarity are also provided. * indicates isolates used in manipulative succession assays.

Day 1	Day 14	Affiliation	Closest taxon (GenBank accession #)	% similarity	*D. pulchra* clone (Genbank accession #)	% similarity
ESS-07	LSS-08	*Gammaproteobacteria*	*Acinetobacter beijerinckii* CIP 110307 (NZ_KB849765.1)	98		
ESS-26		*Actinobacteria*	*Micrococcus luteus* NCTC 2665 (NC_012803.1)	99		
ESS-03	LSS-07	*Actinobacteria*	*Bacillus hwajinpoensis* strain SW-72 (NR_025264.1)	99		
ESS-20		*Alphaproteobacteria*	*Sulfitobacter noctilucae* strain (NR_134205.1)	99		
ESS-08		*Alphaproteobacteria*	*Photobacterium phosphoreum* strain (NR_114184.1)	98		
ESS-23*		*Alphaproteobacteria*	*Thalassobius aestuarii* strain DSM 15283, (NZ_FOTQ01000010.1)	99	*D. pulchra* clone DPC189 (DQ269086)	94
ESS-24*		*Alphaproteobacteria*	*Altererythrobacter ishigakiensis* strain NBRC 107699 (NZ_CP015963.1)	98	*D. pulchra* clone DPC056 (DQ269075)	98
ESS-12		*Bacteroidetes*	*Ornithobacterium rhinotracheale* DSM 15997 (NC_018016.1)	92		
ESS-06*		*Actinobacteria*	*Kytococcus sedentarius* DSM 20547 (NC_013169.1)	99		
ESS-11	LSS-14	*Alphaproteobacteria*	*Tateyamaria omphalii* strain MKT107 (NR_125446.1)	99		
ESS-16		*Bacteroidetes*	*Dokdonia donghaensis* DSW-1 (NZ_CP015125.1)	99	*D. pulchra* clone DPC072 (DQ269097)	94
	LSS-12	*Bacteroidetes*	*Microbulbifer variabilis* ATCC 700307 (NZ_AQYJ01000002.1)	99		
ESS-01	LSS-03	*Gammaproteobacteria*	*Photobacterium gaetbulicola* Gung47 (NZ_CP005974.1)	97		
ESS-05		*Gammaproteobacteria*	*Microbulbifer pacificus* strain SPO729 (NR_115928.1)	98		
	LSS-04	*Gammaproteobacteria*	*Microbulbifer variabilis* strain Ni-2088 (NR_041021.1)	98		
ESS-14	LSS-01	*Alphaproteobacteria*	*Ruegeria atlantica* strain CECT 4292 (NZ_CYPU01000071.1)	99		

ESS-18		*Alphaproteobacteria*	*Pseudovibrio ascidiaceicola* strain DSM 16392 (NZ_FOSK01000033.1)	99		
	LSS-21	*Alphaproteobacteria*	*Labrenzia aggregata* strain RMAR6-6 (NZ_CP019630.1)	99		
	LSS-09*	*Alphaproteobacteria*	*Phaeobacter gallaeciensis* DSM 26640 (NC_023137.1)	99		
	LSS-26	*Alphaproteobacteria*	*Paracoccus beibuensis* strain JLT1284 (NR116400.1)	99		
	LSS-17	*Gammaproteobacteria*	*Tateyamaria omphalii* strain DOK1-4 (NZ_CP019312.1)	98		
	LSS-27	*Actinobacteria*	*Agrococcus lahaulensis* DSM 17612 (NZ_KE384303.1)	99		

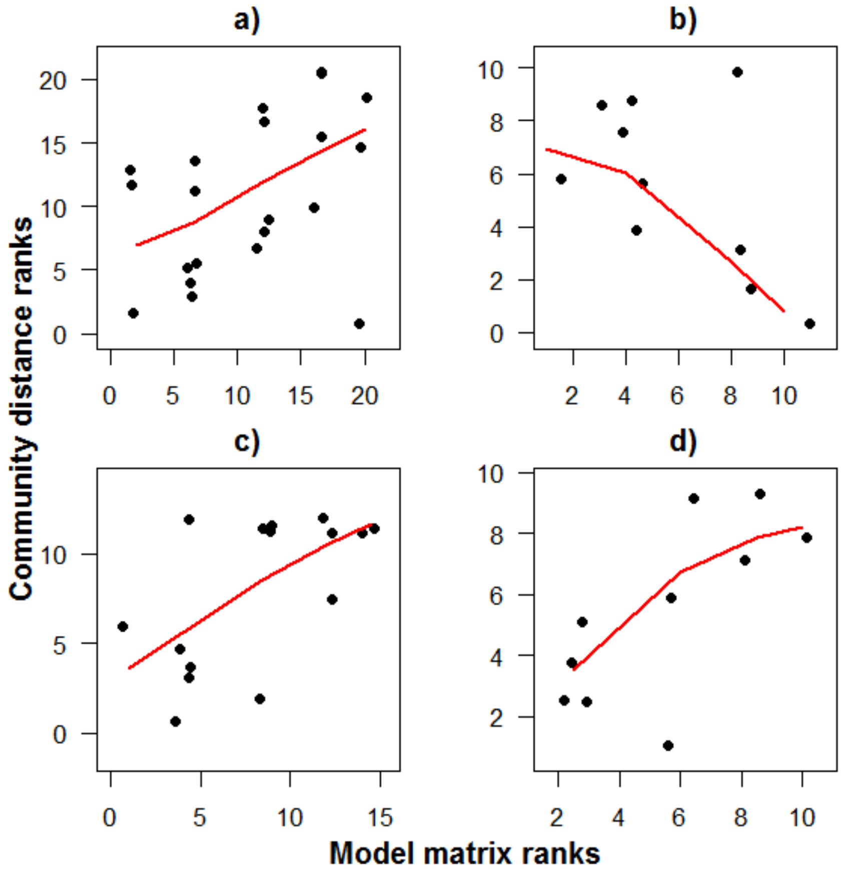

Figure S1. Scatterplot of the rank distances between a cyclic model and community distances (Bray-Curtis similarities) associated with *D. pulchra* whose microbiomes had been experimentally disturbed with a) antibiotic treatments, b) unmanipulated, and microbiomes associated with c) the surrounding seawater, and those associated with d) inanimate surfaces (deployed at the same time as the experimental algae) over time (0-12 d) average among five independent ‘disturbance’ experiments. Results of Mantel tests examining these relationships are given in Table 2.
